# Facilely controlled synthesis of a core-shell structured MOF composite and its derived N-doped hierarchical porous carbon for CO_2_ adsorption†

**DOI:** 10.1039/c8ra03349h

**Published:** 2018-06-12

**Authors:** Zhongzheng Zhang, Nannan Sun, Wei Wei, Yuhan Sun

**Affiliations:** CAS Key Laboratory of Low-Carbon Conversion Science and Engineering, Shanghai Advanced Research Institute, Chinese Academy of Science Shanghai 201203 China weiwei@sari.ac.cn zhangzz@sari.ac.cn; School of Physical Science and Technology, Shanghai Tech University Shanghai 201210 China

## Abstract

A new strategy for controlled synthesis of a MOF composite with a core–shell structure, ZIF-8@resorcinol–urea–formaldehyde resin (ZIF@RUF), is reported for the first time through *in situ* growth of RUF on the surface of ZIF-8 nanoparticles *via* an organic–organic self-assembly process by using hexamethylenetetramine as a formaldehyde-releasing source to effectively control the formation rate of RUF, providing the best opportunity for RUF to selectively grow around the nucleation seeds ZIF-8. Compared with the widely reported method for MOF composite synthesis, our strategy not only avoids the difficulty of incorporating MOF crystals into small pore sized materials because of pore limitation, but also effectively guarantees the formation of a MOF composite with a MOF as the core. After carbonization, a morphology-retaining N-doped hierarchical porous carbon characterized by its highly developed microporosity in conjunction with ordered mesoporosity was obtained. Thanks to this unique microporous core–mesoporous shell structure and significantly enhanced porosity, simultaneous improvements of CO_2_ adsorption capacity and kinetics were achieved. This strategy not only paves a way to the design of other core–shell structured MOF composites, but also provides a promising method to prepare capacity- and kinetics-increased carbon materials for CO_2_ capture.

## Abstract 2

New strategy for controlled synthesis of core–shell structured ZIF-8 composite and hierarchical N-doped carbon *via* an effective *in situ* self-assembly process.

## Graphical abstract

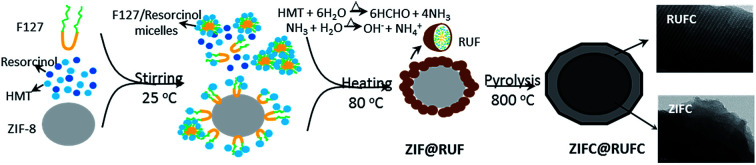

## 1. Introduction

Metal–organic frameworks (MOFs) have attracted tremendous attention and have intriguing potential applications in gas storage,^1–3^ catalysis,^4,5^ optoelectronic devices,^6,7^ and separation^8,9^ because of their high surface area, high adsorption affinity, and diverse structures with various pore shapes, sizes, volumes, and surface chemistry owing to the diversity of metallic centers and organic ligands. However, the weak stability of most MOFs upon exposure to water and high temperature and the difficult shaping of fine powders of synthesized MOFs limit their practical applications.^10–13^ As one of the most promising adsorbents for CO_2_, MOFs have shown more excellent adsorption and separation capability than zeolites, activated carbons, and mesoporous silica/carbon.^14–17^ Unfortunately, the porosity of MOFs is often not fully utilized during adsorption process due to the weak interactions between pore walls and gaseous adsorbates owing to their fully open pore space and low density of atoms in structure.^18–20^ Considering these drawbacks, hybridizing MOFs with porous supports such as silica,^21^ carbon nanotubes,^22–24^ carbon nanofibers,^25^ graphite oxide,^18,26^ mesoporous carbon,^27^ and activated carbon^28^ have been reported to result in new and/or modified properties to circumvent these limitations. These MOFs composites indeed overcome some drawbacks of MOFs and provide better properties due to the existence of synergies between the individual components, such as improved hydrothermal stability, thermal stability and adsorption capacity and selectivity.

Until now, a wide variety of methods for the preparation of metal nanoparticle/MOF composites have been developed, such as solution infiltration method, vapor deposition and solid grinding.^29−31^ However, as far as combining traditional porous materials with MOFs into one construct is concerned, there is almost only one way reported in the literatures, that is, immersion of pre-synthesized porous substrates into the MOF precursor solution, followed by procedures that promote MOFs growth in the cavity and/or on the surface of the porous supports.^21–28^ However, the particle sizes of MOFs (generally from hundreds of nanometers to several microns) are far much larger than the pore sizes of most porous supports, therefore, MOFs generally tend to grow on the outer surface of porous materials rather than in the restricted pore channels, resulting in the formation of MOF composites with supports incorporated into MOFs (Support @ MOF),^19,23,32–34^ that is, MOFs are still exposed, and the hybrid porous materials couldn't offer an effective protection effect for improving MOFs hydrothermal stability. In some worse cases, the so-called MOF composites are actually physical mixtures, in which MOFs and porous supports existed separately due to lack of proper chemical interactions,^27,28,35,36^ and thus the hybrid materials were not shown to exhibit any combined characteristics of the two components. Recently, X. C. Chen *et al.* and P. Pachfule *et al.* reported the incorporation of MOF-5 particles into functionalized nanotubes and nanofibers,^22,25^ respectively, using the above mentioned method. However, the nanotube and nanofiber substrates required pre-functionalization in highly concentrated HNO_3_, making the synthesis more complicated. Besides, the MOF-5 particles are not exclusively confined into the porous pores of substrates in these two systems. In other words, the obtained MOF-5 composites are actually the MOF @ Support @ MOF composites. Although the successful and exclusive doping of MOF-5 into mesoporous SBA-15 without pre-functionalization has been recently reported,^21^ unevenly incorporation of MOF-5 into SBA-15 are obtained, more seriously, the provided evidences for supporting the incorporation of MOF-5 into SBA-15 pore channels are not sufficient because the observed Zn signal in the EDX scans of mesoporous region of SBA-15 is likely to originate from the adsorbed Zn^2+^ ions by hydroxyl groups of SBA-15 and/or the basic structural building units of Zn_4_O. Therefore, a facile and highly effective strategy for fabricating MOF composites with MOF exclusively embedded in porous supports (MOF @ Support) is urgently desired.

Alternatively, MOFs were recently employed as precursors for fabrication of porous carbon materials. For the type of Zn-based MOFs, ZnO generated from pyrolysis of Zn-based MOFs, well-known as a carbon activation agent, plays an important role of *in situ* self-activation during conversion of MOF to carbon materials at high temperatures. At the same time, metallic Zn generated *via* reduction by carbons subsequently evaporates, which may play a potential role in the formation of additional porosity. This is to say that if a “soft” material, such as polymers, can be well-designed to coat on Zn-based MOF particles to form MOF@polymer composites, the escaped Zn from inside will further pass through shell during their carbonization process, inevitably creating new pores in the shell material. Therefore, attaching a polymer onto the surface of Zn-based MOFs to form MOF@Support composite is a good design for the preparation of highly porous carbon materials with enhanced porosity.

Herein, taking the synthesis of core–shell structured ZIF-8@resorcinol–urea–formaldehyde resin composite (ZIF@RUF) as an example, we present a new and universal synthetic strategy for the preparation of MOF composites for the first time. Unlike most of the reported literatures, our approach relies on the direct introduction of RUF precursors to the solution containing highly dispersed ZIF-8 particles to allow *in situ* polymerization and condensation of RUF resin on the surface of ZIF-8 particles *via* an organic–organic self-assembly method. To achieve the precise encapsulation of ZIF-8 into RUF shell material, a slow releasing source of formaldehyde, hexamethylenetetramine, was rationally employed to avoid the formation of RUF in the bulk solution, thereby providing the best opportunity for RUF growth around ZIF-8 particles as nucleation seeds to form MOF composites with ZIF-8 as the exclusive core. In addition, a morphology-preserved hierarchically porous N-doped carbon material (ZIFC@RUFC) with microporous core and mesoporous shell and significantly enhanced CO_2_ capture performance than the individual counterparts can be directly derived from the obtained ZIF@RUF composite through a simple carbonization process under N_2_ atmosphere, which demonstrates a promising way to prepare porosity-enhanced porous carbon materials by employing the *in situ* self-activation of ZnO nanoparticles and the escape of Zn atoms.

## 2. Experimental

### 2.1 Chemicals

Triblock copolymer Pluronic F127 (*M*_w_ = 12 600) was purchased from Sigma-Aldrich Co. 2-Methylimidazole (98%), zinc nitrate hexahydrate (99.99%), resorcinol (AR), hexamethylenetetramine (HMT, 99.0%), urea (AR), and ammonia solution (28–30%) were supplied by Aladdin Industrial Co. All chemicals were used as received without further purification.

### 2.2 Synthesis procedures

ZIF-8 was synthesized according to the following procedure: 0.733 g of Zn(NO_3_)_2_·6H_2_O was dissolved in 20 mL of deionized water, and 6.488 g of 2-methylimidazole was dissolved in 80 mL of water separately. The above two solutions were mixed and aged at room temperature for 2 h. After 2 h, white ZIF-8 particles were collected by filtration, washed with water, and dried at 80 °C for 24 h. ZIF-8-derived carbon material (denoted as ZIFC) was obtained by carbonizing ZIF-8 particles at 800 °C for 5 h with a heating rate of 2 °C min^−1^ under nitrogen atmosphere. Char yield (CY) of ZIF-8 is about 50.1 wt%.

RUF resin was prepared by a self-assembly route according to previous publication.^37^ In a typical synthesis, 2.2 g of F127, 1.1 g of resorcinol, 0.7 g of HMT, and 2.0 mL of ammonia solution were mixed in 54 mL of deionized water. After stirring for 1 h at room temperature, 0.6 g of urea and 0.35 g of HMT were added. The obtained solution was further treated at 80 °C for 24 h. Finally, the solid products were collected by filtration, washed with water, and dried at 100 °C for 24 h. RUF resin-derived carbon (denoted as RUFC) was also obtained following similar carbonization procedure to that of ZIF-8 with a CY of *ca.* 23.7 wt%.

**Fig. 1:**
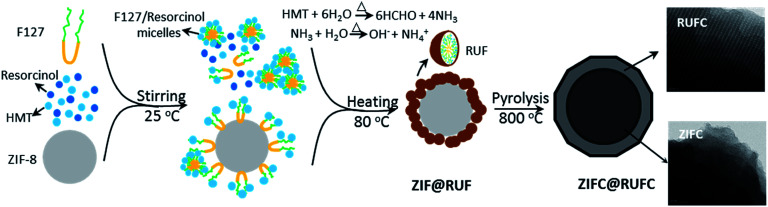
Synthesis of ZIF@RUF and ZIFC@RUFC.

For the synthesis of ZIF@RUF, 0.35 g of ZIF-8 particles was dispersed into 54 mL of deionized water through stirring and ultra-sonication. Afterwards, 2.2 g of F127, 1.1 g of resorcinol, 0.7 g of HMT, and 2.0 mL of aqueous ammonia were added into the ZIF-8-containing solution. After 1 h stirring, 0.6 g urea and 0.35 g HMT were added, and then the final solution was aged at 80 °C for 24 h to complete the *in situ* condensation, polymerization, and growth of RUF resin on the surface of ZIF-8. ZIF@RUF composite was obtained after filtration, washing, and drying. After carbonization of ZIF@RUF composite at 800 °C for 5 h under nitrogen atmosphere, ZIF@RUF-derived carbon, referred to ZIFC@RUFC, was further obtained with a CY of 23.7 wt%. The schematic fabrication process of ZIF@RUF and ZIFC@RUFC are illustrated in Fig. 1.

For comparison, a physical mixture of ZIF-8 and RUF, denoted as ZIF-RUF, was also prepared by thoroughly mixing and grinding ZIF-8 and RUF with identical composition of ZIF@RUF.

### 2.3 Characterization

N_2_ sorption isotherms were measured at 77 K on Micromeritic ASAP 2040. Before sorption measurement, sample was degassed at 250 °C for 24 h under vacuum to remove any adsorbed impurities. The specific surface area (*S*_BET_) and total pore volume (*V*_t_) was calculated by using the Brunauer–Emmett–Teller (BET) method and the adsorbed amount of N_2_ at relative pressure >0.995, respectively, while the micropore area (*S*_mic_) and micropore volume (*V*_mic_) were estimated by the *t*-plot method, and the pore size distribution (PSD) curves were obtained by the none local density functional theory (NLDFT) with slit and cylindrical geometries. X-ray diffraction (XRD) patterns were collected using a Bruker D8 diffractometer, and morphology and structure of the obtained samples were investigated by scanning electron microscopy (SEM) on a SUPRRA 55 equipment. Transmission electron microscopy (TEM) was performed on JEOL 2100F. Nitrogen content and surface chemistry of N-doped samples were measured by CHN analyzer and X-ray photoelectron spectroscopy (XPS) technique using a quantum 2000 electron spectrometer. CO_2_ sorption isotherms at 25 °C from 0 to 1 bar were conducted on a Micromeritics ASAP 2020 instrument, while CO_2_ adsorption kinetics at 35 °C and 1 bar were carried out on a TGA Q50 thermal gravimetric analyzer as follows: *ca.* 20 mg of sample was loaded in a platinum pan, heated at 300 °C for 3 h with a heating rate of 5 °C min^−1^ under a He atmosphere with 100 mL min^−1^. When the temperature was cooled to the target temperature (35 °C in our study), CO_2_ was immediately introduced to the sample by switching He to CO_2_ until complete adsorption saturation. Besides, the thermal analysis of samples was performed using a thermogravimetric analyzer (STA 449 F3, Netzsch) in N_2_ atmosphere with a heating rate of 5 °C min^−1^.

## 3. Results and discussion

**Fig. 2:**
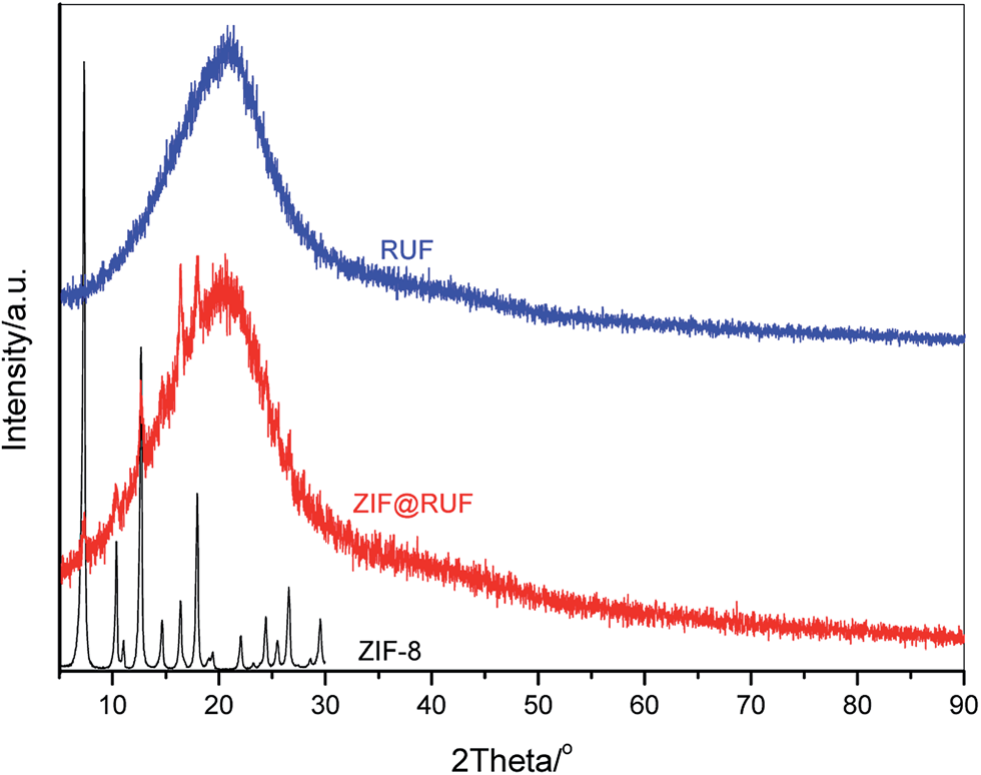
Wide angle XRD patterns of ZIF-8, RUF and ZIF@RUF.

Fig. 2 shows wide angle XRD patterns of the as-prepared ZIF-8, RUF, and their composite ZIF@RUF. ZIF-8 shows the same characteristic peaks with the simulated ZIF-8.^38^ The well-resolved peaks imply the high crystallinity of the ZIF-8 sample obtained in this work, while RUF is an amorphous material indicated by the exclusive and broad peak at *ca.* 21°. As expected, ZIF@RUF sample shows both peaks characteristic for ZIF-8 and RUF, indicating the formation of a hybrid material. Although the physical mixture ZIF-RUF also exhibits a similar XRD pattern to that of ZIF@RUF (Fig. S1†), the considerably stronger intensity of ZIF-RUF suggests distinctive structures. In the subsequent study, it was confirmed that the lower intensity of ZIF-8 characteristic peaks in ZIF@RUF was caused by the complete encapsulation of ZIF-8 into the RUF frameworks.

**Fig. 3:**
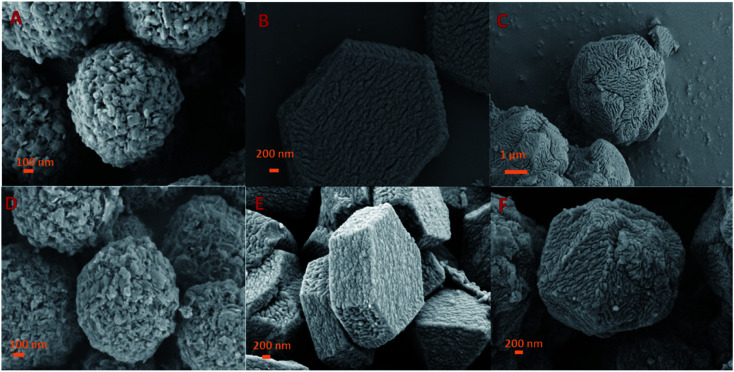
SEM images of ZIF-8 (A), RUF (B), and ZIF@RUF (C), and their corresponding carbonized sample ZIFC (D), RUFC (E), and ZIFC@RUFC (F).

To investigate the structural variation of the samples, SEM images were collected. As shown in Fig. 3A and S2,† ZIF-8 particles obtained in our study showed an spherical morphology with uniform diameters of *ca.* 1–2 μm, meanwhile, a rough outer surface along with an interconnected reticular inner structure were observed. It can be seen that the ZIF-8 spheres are composed of ZIF-8 nano-sheets, which can be evidenced from TEM images of the small fragments in Fig. S2.† SEM images of RUF in Fig. 3B and S3† showed that RUF particles possess a uniform prism structure of several micrometers in size. Besides, on the surface of RUF sample, regularly sulcate stripes are clearly visible, which may be indicative of regular alignment of pore structures in RUF. Interestingly, after incorporating of ZIF-8 into RUF, the ZIF@RUF composite unexpectedly showed an entirely different morphology from the individual counterparts of ZIF-8 and RUF. As shown in Fig. 3C, ZIF@RUF composite exhibited a polyhedral spherical shape. This shape is somewhat similar to that of ZIF-8 particles, but still with some difference and the surface of ZIF@RUF with some visible deep stripes is extremely similar to that of RUF. Thus, we preliminary expected that ZIF-8 particles were embedded into RUF so as to form the unique morphology with the similar shape of ZIF-8 and the similar surface of RUF. Further investigations of ZIF@RUF SEM images in Fig. S4† revealed that ZIF-8 spheres cannot be found any more from the panoramic view, this demonstrates that all the ZIF-8 particles were totally embedded into RUF. These results convinced us that the *in situ* growth of RUF on the surface of ZIF-8 spheres results in the encapsulation of ZIF-8 by the RUF frameworks and lead to the formation of a core–shell structure: ZIF-8 serves as the core and the RUF serves as the shell. Due to the weak textural properties of RUF and ZIF@RUF (*S*_BET_: 0.25 and 0.12 m^2^ g^−1^, respectively), carbonization of RUF and ZIF@RUF at high temperature 800 °C were carried out to remove the template F127 to further activate the occupied pores. As shown in Fig. 3D, E, and F, after the high temperature thermal conversion to carbon, the original morphology and shape of ZIF-8, RUF, and ZIF@RUF samples were well retained in their corresponding carbon materials, indicating good morphology stability of ZIF@RUF, ZIF-8, and RUF samples.

**Fig. 4:**
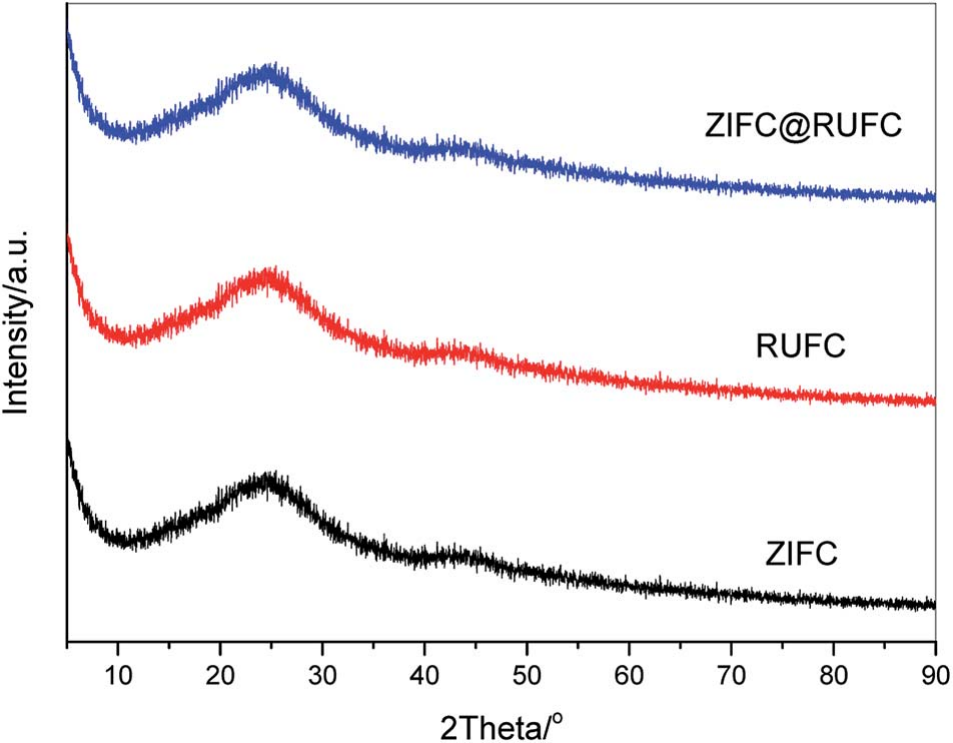
Wide angle XRD patterns of ZIFC, RUFC and ZIFC@RUFC samples.

Fig. 4 reveals the wide-angle XRD patterns of the carbonized samples. It is clearly that after carbonization at 800 °C in N_2_ atmosphere, similar patterns were obtained for all the samples, where one broadened reflection at 2*θ* = 24° and another relatively weak peak at 2*θ* = 44° were found, these peaks can be assigned to the (002) and (100) reflection planes of disordered graphitic carbon, respectively.^39,40^ Interestingly, no characteristic diffraction peaks of Zn-related species, originated from the thermal decomposition of ZIF-8, are visible in the patterns of ZIFC and ZIFC@RUFC. This might be because most of the generated ZnO from the pyrolysis of ZIF-8 are small in size and bearing high reactivity, therefore could be easily reduced by carbon into metallic Zn (ZnO + C = Zn + CO), the latter partially evaporates at the high temperature (800 °C) due to its low boiling point (907 °C).^41^ This explanation can be confirmed from the XRD pattern (Fig. S5†) of a comparison sample ZIFC-600 prepared by pyrolyzing ZIF-8 at 600 °C, where very distinct and high-intensity characteristic peaks of ZnO are observed. On the other hand, it should be noted that there are still trace amount of residual ZnO particles left in the inner pores. However, the amount is too low to be detected by XRD because of the deep encapsulation by carbon, which was subsequently supported by TEM and XPS analysis.

**Fig. 5:**
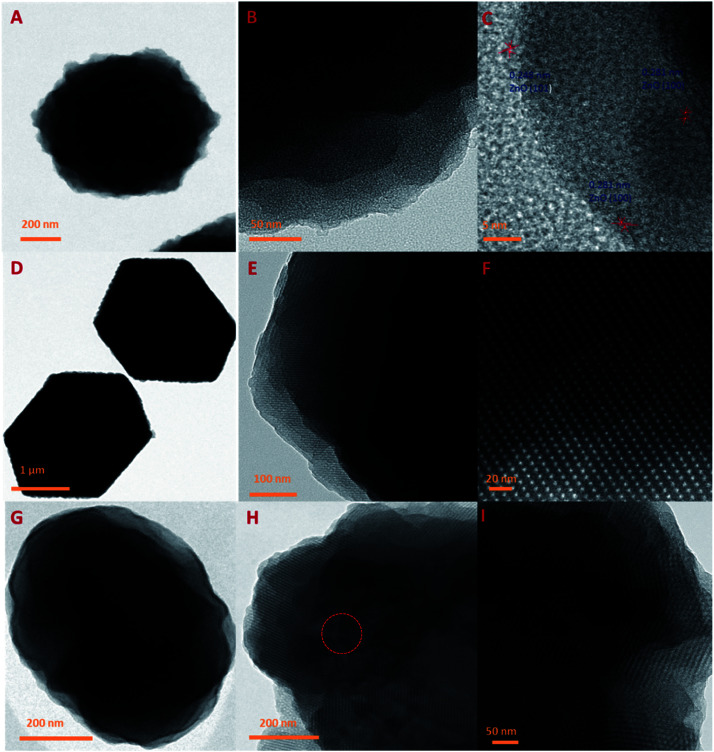
TEM images of ZIFC (A–C), RUFC (D–F) and ZIFC@RUFC (G–I).

Fig. 5 represents the TEM images of ZIFC, RUFC, and the ZIFC@RUFC composite. All these samples inherit the nanoarchitecture of their precursors (Fig. 5A, D, and G). As shown by TEM images of ZIFC in Fig. 5B and C, amorphous porous carbon was generated through the pyrolysis of ZIF-8, and meanwhile, trapped ZnO domains also existed in the porous carbon matrices with the interplanar spacing of 0.248 nm and 0.281 nm, which can be indexed as the (101) and (100) lattice spacing of ZnO (JCPDS no. 89-1397), respectively. This result, indicating the presence of ZnO in ZIFC, combined with the analysis result of the XRD pattern of ZIFC where no characteristic peaks of ZnO were observed, indicated that the content of ZnO in ZIFC sample is very low, and demonstrated again that most of the generated ZnO species were reduced by carbon into evaporable Zn atoms at high temperature, and only trace amounts of ZnO particles survived due to strong encapsulation by carbon frameworks. For the RUFC sample, highly ordered arrangement of porous structure is clearly observed similar to previous literatures (Fig. 5E and F),^42,43^ this can be attributed to the controlled release of formaldehyde by HMT, which in turn influenced the condensation/polymerization kinetics of resorcinol-urea-formaldehyde resin (RUF). In Fig. 5H and I, ordered stripe-like pore arrangements are also observed in the TEM images of ZIFC@RUFC, which can be reasonably related to the mesoporous structure of RUFC, as we demonstrated in the above that all the ZIF-8 particles were fully coated by RUF. Together with the TEM images of ZIFC@RUFC displayed in Fig. S6,† the above results further demonstrated the core/shell structure of ZIFC@RUFC composite.

**Fig. 6:**
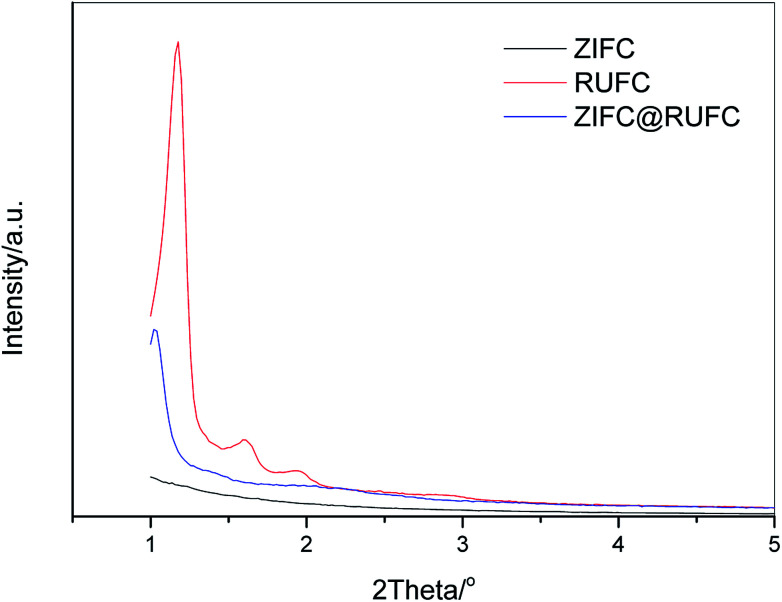
Small angle XRD patterns of ZIFC, RUFC, and ZIFC@RUFC.

In order to further investigate the mesostructure of RUFC and ZIFC@RUFC, small angle XRD measurements were carried out. Fig. 6 depicts the patterns of RUFC and ZIFC@RUFC samples as well as the sample ZIFC as a comparison. Due to the disordered amorphous nature of ZIFC, no diffraction peaks were observed for the ZIFC sample. For RUFC, three well-resolved peaks (2*θ* = 1.18°, 1.61° and 1.94°) and one weak peak (2*θ* = 2.45°) were obtained, indicating again the highly ordered mesostructure of RUFC. The reciprocal d-spacing values of these reflection peaks follow the relationship of 
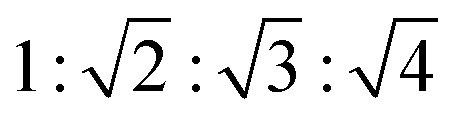
, and thus can be indexed as (110), (200), (211) and (220) reflections, associated with the body-centered cubic structure, similar to the early reports.^37,42,43^ Similarly, the ZIFC@RUFC sample also possesses the ordered mesostructure, indicated by one well-shaped reflection peak (2*θ* = 1.03°) and three weak peaks (2*θ* = 1.44°, 2.06°, and 2.23°). However, the significant decrease in peaks intensity of the ZIFC@RUFC sample indicates that the mesostructure of ZIFC@RUFC is less ordered than that of RUFC sample probably due to the presence of ZIFC core. Moreover, the corresponding peaks are shifted to lower angle, implying incorporation of ZIF-8 into RUF frameworks caused a substantial expansion of the mesostructures of RUFC, which coincides with the pore size diameter of RUFC (3.6 nm) and ZIFC@RUFC (4.7 nm) estimated from TEM images.

**Fig. 7:**
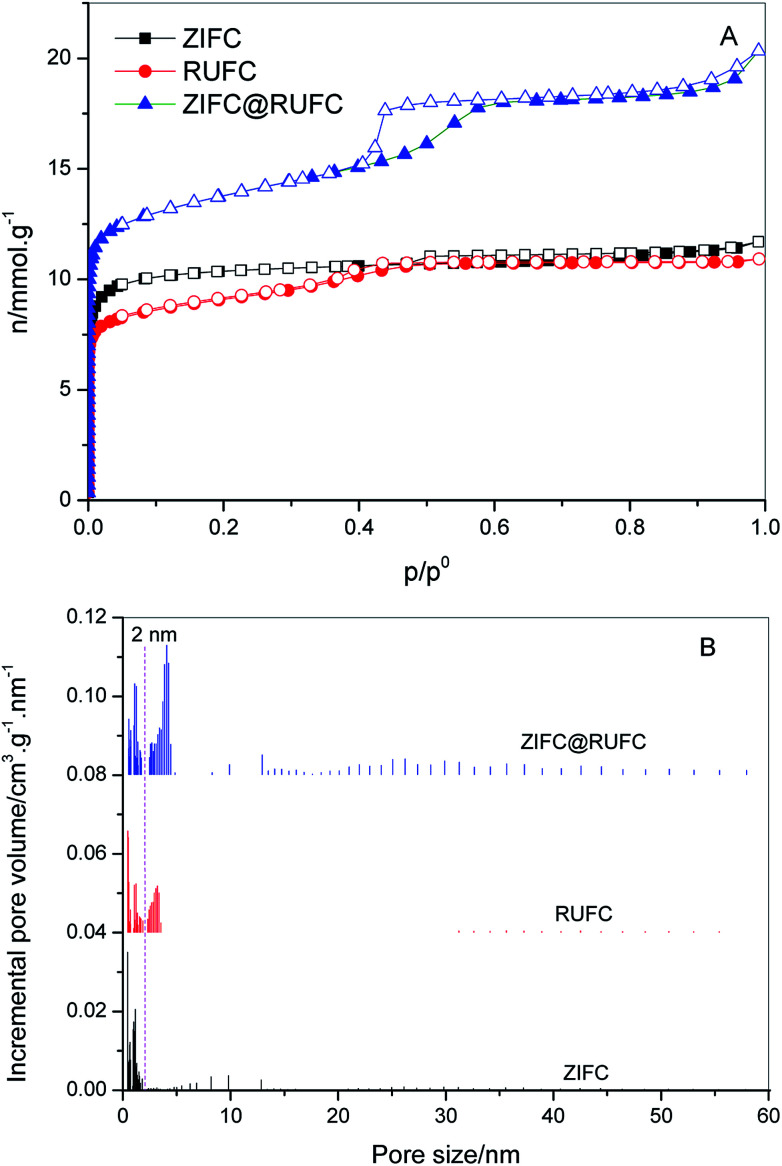
Nitrogen sorption isotherms (A) and NLDFT pore size distributions (B) of ZIFC, RUFC, and ZIFC@RUFC. The pore size distributions are offset vertically by 0.04 and 0.08 cm^3^ g^−1^ nm^−1^ for RUFC and ZIFC@RUFC, respectively.

To assess the textural properties of the prepared carbons, N_2_ adsorption–desorption isotherms were acquired and summarized in Fig. 7A. A type-I isotherm was observed for ZIFC, implying its microporous structure, meanwhile, the co-existence of minor mesoporosity can be confirmed by the noticeable hysteresis, probably arising from the voids formed after the removal of ZnO. These results are identical with its corresponding pore size distribution curve (Fig. 7B), where ZIFC is characterized by an overwhelming majority of micropores together with a few mesopores. Different from ZIFC, RUFC shows a type-IV isotherm with a clear capillary condensation steps and an obvious H2-type hysteresis loop at the region of *p*/*p*^0^ = 0.4–0.5, corresponding to a mesostructure with a narrow mesopore distribution. At the same time, a sharp increase of N_2_ uptake at low pressures, comparable to that of ZIFC, indicates the highly developed micropores in the RUFC matrix. The corresponding PSD of RUFC in Fig. 7B reveals several maxima in the micropore range below 2 nm and one narrow peak from the primary mesopores with 3.3 nm, which is in good agreement with the estimated mesopore size value of RUFC (3.6 nm) from the TEM images. The micropores are assumed to be generated by both the pyrolysis of the resin frameworks and the removal of embedded PEO segments from the pore walls, while the mesopores were generated from the decomposition of surfactant F127. ZIFC@RUFC exhibited typical type-IV curves similar to that of RUFC as can be reasonably deduced, implying the mesoporous characteristic of the sample. However, unexpectedly, compared with the individual counterparts, the N_2_ uptake of ZIFC@RUFC is dramatically higher, indicating the significantly enhanced textural properties such as specific surface area and pore volume. For example, as listed in Table 1, the ZIFC@RUFC composite exhibited a high surface area of 982 m^2^ g^−1^, increased by 38% and 52% as compared with ZIFC and RUFC, respectively. Similarly, the total pore volume was 73% and 84% higher than that of ZIFC and RUFC samples, respectively. This reveals the presence of a remarkable synergetic effect between the two components of the composite. From a theoretical point of view, it is interesting to compare the measured values of the composite structural parameters with a “hypothetical” mixture, namely the physical mixture of ZIFC and RUFC. These “hypothetical” parameters can be calculated according to eqn (1)–(4) in the following:

1 *X*_*n*_ = *X*_ZIFC_ × wt_ZIFC_% + *X*_RUFC_ × wt_RUFC_%

2
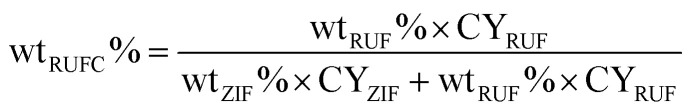

3 wt_ZIF_% = 1 − wt_RUF_%

4 wt_ZIFC_% = 1 − wt_RUFC_%

where *X*_ZIFC_, *X*_RUFC_, and *X*_*n*_ are any parameters such as surface area, pore volume, micropore surface area and micropore volume for ZIFC, RUFC, and the “hypothetical” mixture, respectively. wt_ZIFC_% and wt_RUFC_% are the weight percentage of ZIFC and RUFC in the composite ZIFC@RUFC, wt_ZIF_% and wt_RUF_% represent the weight percentage of ZIF-8 and RUF in the ZIF@RUF composite, and CY_ZIF_ and CY_RUF_ are the char yield of ZIF-8 and RUF, which were calculated as 50.1 wt% and 23.7 wt%, respectively (Experimental section). wt_RUF_% can be estimated based on the ratio of the weight loss of dried ZIF@RUF to the weight loss of the dried RUF in the temperature range of 255–450 °C, since ZIF-8 shows almost no weight loss at this temperature range (Fig. S7†). The weight loss of dried RUF and ZIF@RUF from 255 °C to 450 °C is about 55.44 wt% and 50.23 wt%, respectively. Thus, the weight content of RUF in ZIF@RUF is determined to be 90.6 wt%, and the weight percentage of RUFC in ZIFC@RUFC sample is calculated to be 82.4 wt%. Based on the above assumption, the calculated parameters for the “hypothetical” mixture are presented in Table 1. It is clear that the measured values are always higher than those calculated for the “hypothetical” mixture, this indicates once more the synergy between the individual components of the hybrid ZIFC@RUFC. We also compared the PSD of ZIFC@RUFC with ZIFC and RUFC. As shown in Fig. 7B, ZIFC@RUFC displays obvious widening of mesopore diameters (4.1 *vs.* 3.3 nm) probably due to the increasing of d-spacing of RUF as indicated by the small angle XRD. Besides, enhanced mesoporosity (mainly above 10 nm) was also observed on ZIFC@RUFC, which can be attributed to the evaporation of metallic Zn and/or carbon activation by ZnO since ZIFC@RUFC and ZIFC possessed similar PSD in the range above 5 nm.

**Table 1:** Textural properties of ZIFC, RUFC and ZIFC@RUFC composite

Sample	*S*_BET_ a/m^2^ g^−1^	*S*_mic_ b/m^2^ g^−1^	*V*_t_ c/cm^3^ g^−1^	*V*_mic_ b/cm^3^ g^−1^	*D* d/nm	N content/%	
						CHN	XPS
ZIFC	713	603	0.41	0.31	—	7.52	7.12
RUFC	647	411	0.38	0.21	3.3	1.32	1.37
ZIFC@RUFC	982(659)^e^	612(445)^e^	0.70(0.39)^e^	0.32(0.23)^e^	4.1	1.39	1.49

a Surface areas calculated using the BET equation in the relative pressure range of 0.05–0.30.

b Microporous surface areas and micropore volumes calculated using the *t*-plot method in the thickness range of 0.4–0.6 nm.

c Single point total pore volumes determined from the N_2_ uptakes at relative pressures >0.995.

d Mesopore diameters at the maxima of PSD curves.

e The numbers in brackets are the correspondingly “hypothetical” textural parameters.

On the basis of the above observations, a possible growth and encapsulation mechanism for the current approach to synthesize MOF composites is proposed (Fig. 1). First, the resorcinol molecules with a high hydroxyl density can strongly interact with the PEO segments of the amphiphilic triblock copolymer F127 *via* H-bonding interactions to form spherical F127/resorcinol composite micelles. Under hydrothermal conditions, the high temperature and autogenous pressure promoted HMT, as a slow releasing source of formaldehyde and PH buffer, to gradually hydrolyze into formaldehyde and ammonia. The slowly released formaldehyde further reacted with resorcinol and urea under the catalysis of ammonia, and then polymerize into RUF resin in a controllable manner. The resulted F127/RUF composite served as a structural building unit, and with further cross-linking of RUF resins, the F127/RUF composite micelles connect with each other through carbon–carbon bonds to form micelle aggregates. These aggregates were eventually adsorbed and attached on the surface of ZIF-8 nuclei due to the adsorption potential energy of ZIF-8 surface, and closely packed to generate rigid structures in a body-centered cubic manner. After carbonization, ZIFC@RUFC composite with well-preserved mesostructure and morphology were generated.

To further investigate the chemical composition of the carbonized samples, XPS analysis was conducted on ZIFC, RUFC, and ZIFC@RUFC. Fig. S8† shows their corresponding XPS survey spectra. All the samples displayed three typical peaks for C 1s (285 eV), N 1s (400 eV), and O 1s (534 eV). In addition, two distinct peaks at *ca.* 1022 and 1045 eV, assigned to Zn 2p, were also observed in ZIFC spectrum, indicating the presence of Zn species. However, due to the fact that the ZnO species are coated by RUFC, visible yet indistinct Zn 2p peaks were detected for ZIFC@RUFC (inset in Fig. S8†). Additionally, the Zn content in ZIFC and composite was determined to be about 1.87 at% and 0.21 at%, respectively (Fig. S9†). These low values are responsible for the missing of ZnO characteristic peaks in the XRD patterns of these samples. The surface nitrogen content determined from XPS is as high as 7.12 at% for ZIFC, while the content of nitrogen in ZIFC@RUFC composite is about 1.49 at%, comparable to that of RUFC (1.37 at%). We also measured the bulk nitrogen content from CHN analyzer (Table 1), similar values were obtained, indicating the decreasing of N content in the ZIFC@RUFC composite, which can be related to the significantly higher concentration of RUFC in composite.

**Fig. 8:**
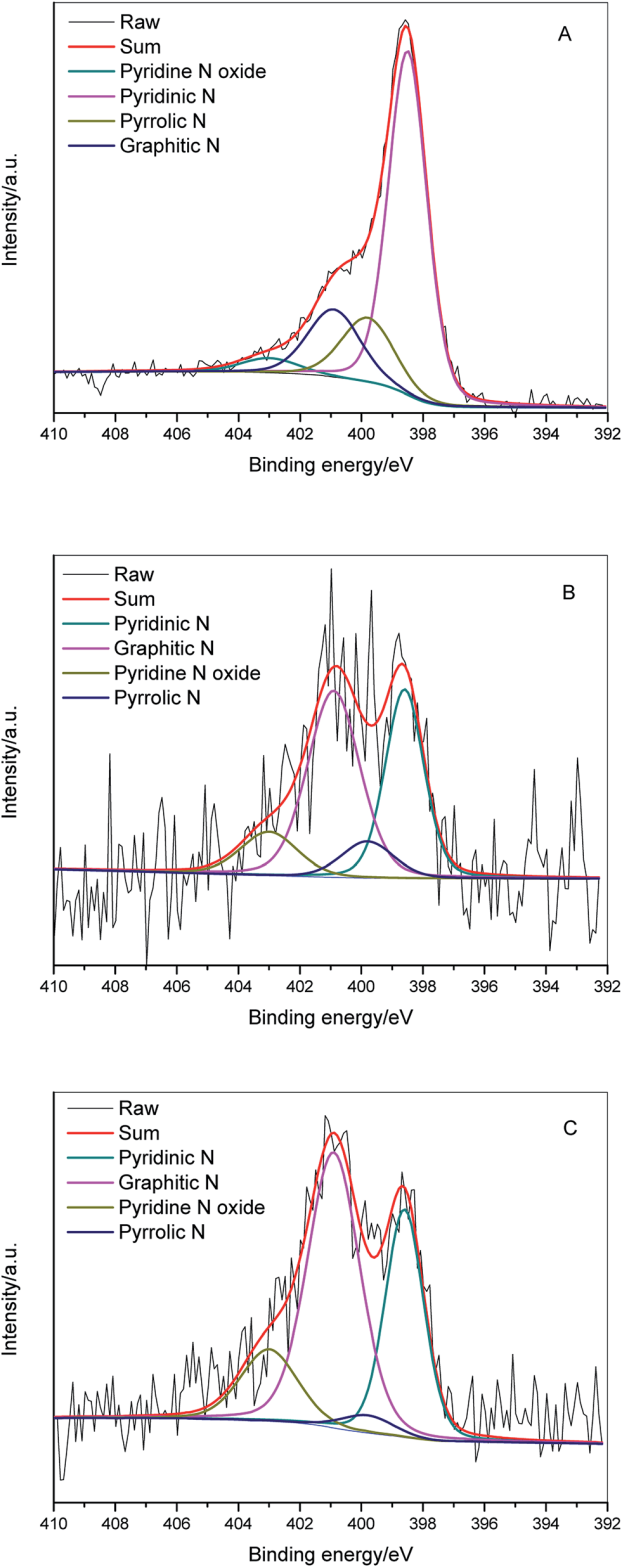
N1s XPS spectra of ZIFC (A), RUFC (B) and ZIFC@RUFC (C).

In order to access additional information about the chemical nature of incorporated nitrogen species, N 1s XPS of ZIFC (Fig. 8A), RUFC (Fig. 8B), and ZIFC@RUFC (Fig. 8C) were fitted into four peaks centered at 398.5 eV, 399.8 eV, 400.9 eV, and 403 eV, which can be assigned to pyridinic N, pyrrolic N, graphitic N and pyridine N-oxide, respectively.^43,44^ The above analysis demonstrated the encapsulation of ZIFC by RUFC in the ZIFC@RUFC composite, therefore, in theory, the surface characteristics of ZIFC@RUFC material should be similar to RUFC. However, as shown in Table 2, different surface concentrations of individual nitrogen species between ZIFC@RUFC and RUFC were observed. This is to say the incorporation of a ZIF-8 core in the ZIF@RUF affected its subsequent carbonization, resulting in altered distribution of different nitrogen species.

**Table 2:** Relative surface concentrations of nitrogen species obtained by fitting the N1s XPS spectra

Sample	Pyridinic N/%	Pyrrolic N/%	Graphitic N/%	Pyridine N oxide/%
ZIFC	63.1	16.0	16.9	4.0
RUFC	34.0	8.9	45.6	11.6
ZIFC@RUFC	31.2	3.3	51.1	14.4

**Fig. 9:**
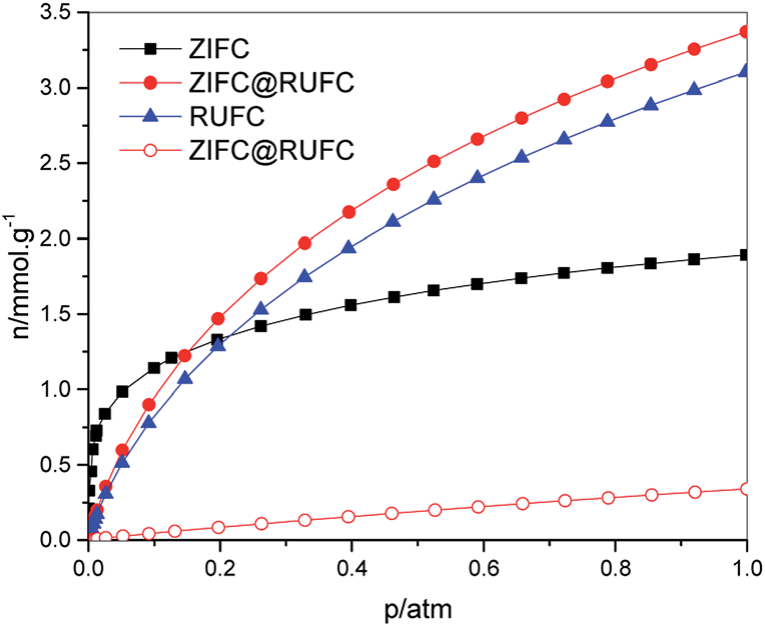
CO_2_ isotherms (solid) of ZIFC, RUFC and ZIFC@RUFC and N_2_ isotherm (open) of ZIFC@RUFC at 298 K.

The highly developed hierarchical porous structure of ZIFC@RUFC encouraged us to investigate its adsorption performance, therefore, CO_2_ adsorption was evaluated and compared with ZIFC and RUFC. CO_2_ adsorption isotherms for ZIFC@RUFC, ZIFC, and RUFC are showed in Fig. 9. At 1 bar, ZIFC@RUFC exhibited the highest CO_2_ capacity of 3.37 mmol g^−1^. Although this value is lower than some N-doped carbon materials, such as SU-MAC-500 and SU-MAC-600 (4.50 and 4.18 mmol g^−1^, respectively),^45^ it is still comparable to that of the nitride OMCs (3.46 mmol g^−1^)^46^ and SU-MAX-800 (3.11 mmol g^−1^),^45^ much higher than that of the nitrogen and magnesium codoped mesoporous carbon composite (2.26 mmol g^−1^),^47^ the hollow carbon spheres with high nitrogen content of 14.8% (2.67 mmol g^−1^),^48^ and the H-NMC-2.5 with high nitrogen content of 13.1% (2.8 mmol g^−1^).^49^ Due to the enhanced surface area and porosity of ZIFC@RUFC, ZIFC@RUFC exhibits higher CO_2_ adsorption capacity (3.37 mmol g^−1^) than ZIFC (1.89 mmol g^−1^) and RUFC (3.10 mmol g^−1^) at 1 bar. However, to our surprise, RUFC, which possesses smaller surface area and porosity than ZIFC, has a much larger capacity than ZIFC. As mentioned above, ZIFC is a mainly microporous material, while RUFC is composed of both mesopores and micropores. It is believed that the large width of mesopores is beneficial for the diffusion of CO_2_ into the inner channels and makes the active sites easily accessible. Similarly, the additional mesopores above 10 nm in ZIFC@RUFC, that increased its surface area and porosity, are also responsible for its higher CO_2_ capacity than RUFC. It is also worth to mentioned that, although ZIFC@RUFC shows a much lower nitrogen content (1.39 wt%) than ZIFC (7.52 wt%), fortunately, ZIFC@RUFC exhibits almost the same low-pressure capacity with ZIFC at 0.15 bar. This can be ascribed to the fact that the increase of textural properties of ZIFC@RUFC compensated the decrease in the nitrogen content. In addition, to evaluate the CO_2_ separation performance of the ZIFC@RUFC composite, N_2_ isotherm on ZIFC@RUFC was also collected at 298 K (Fig. 9). The N_2_ adsorption capacity is about 0.34 mmol g^−1^ at 1.0 bar, which is much lower than that of CO_2_ capacity at the same condition. The initial slopes of the CO_2_ and N_2_ adsorption isotherms are calculated to be 14.97 and 0.75, respectively. Therefore, the selectivity of CO_2_ over N_2_ is about 20 : 1, which is comparable or even better than some N-doped carbons with higher N content than ZIFC@RUFC, such as the hollow carbon spheres with N content of 14.8% (29 : 1)^48^ and the SU-MAC-800 with N content of 3.2% (9 : 1).^45^

**Fig. 10:**
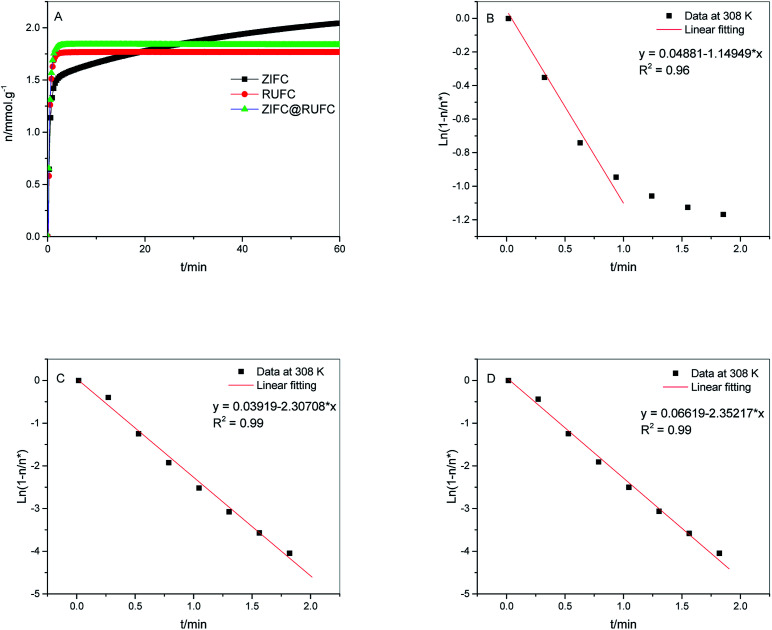
Adsorption kinetics curves of CO_2_ on ZIFC, RUFC and ZIFC@RUFC sample at 308 K and 1.0 bar (A). Linear dependence between ln(1 − *q*/q*) and time for the analysis of CO_2_ adsorption kinetics on ZIFC (B), RUFC (C), and ZIFC@RUFC (D) using the LDF model.

To verify the faster diffusion of CO_2_ on mesoporous carbons (RUFC and ZIFC@RUFC) than microporous ZIFC sample, kinetics of CO_2_ adsorption were carried out by using a TGA Q50 thermal gravimetric analyzer. Adsorption rate of CO_2_ was assumed to follow the linear driving force (LDF) model as follows:5
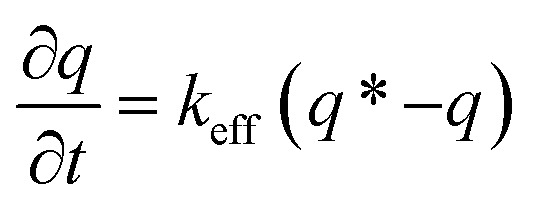
where *k*_eff_ is the mass-transfer constant, q* is the equilibrium CO_2_ uptake, and *q* is the CO_2_ uptake at time *t*. Fig. 10A shows the kinetic curves of CO_2_ adsorption on ZIFC, RUFC, and ZIFC@RUFC at 308 K and 1.0 bar, based on which, fitting with LDF model was carried out as showed in Fig. 10B–D, and the mass-transfer constants for CO_2_ at 308 K and 1.0 bar were estimated to be 0.0192, 0.0385, and 0.0392 s^−1^ for ZIFC, RUFC, and ZIFC@RUFC, respectively. Further, the diffusion coefficient, *D*_*e*_, can be calculated by eqn (6):^50,51^6
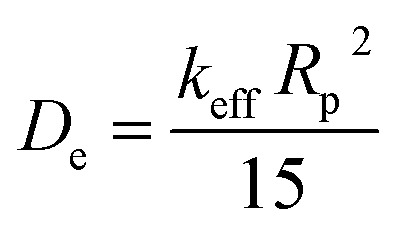
where *D*_e_ (cm^2^ s^−1^) is the interparticle diffusion coefficient, *R*_p_ (cm) is the particle radius, and *k*_eff_ (s^−1^) is the mass-transfer coefficient. As shown in Fig. S10,† the average particle size of ZIFC, RUFC, and ZIFC@RUFC sample is about 1.60, 1.49, and 1.61 μm, respectively. Therefore, according to eqn (6), the diffusion coefficient of CO_2_ is 3.31 × 10^−11^, 5.69 × 10^−11^, and 6.77 × 10^−11^ cm^2^ s^−1^ for ZIFC, RUFC, and ZIFC@RUFC, flowing the trend of ZIFC < RUFC < ZIFC@RUFC, strongly indicating the adsorption of CO_2_ was more rapid on mesoporous carbons RUFC and ZIFC@RUFC.

## 4. Conclusions

A novel hybrid material with core–shell structure, ZIF@RUF, has been successfully synthesized for the first time from a new synthetic strategy, which relies on the *in situ* growth and polymerization of RUF polymers *via* an organic–organic self-assembly process on the surface of ZIF-8 nanoparticles by using HMT as a release source of formaldehyde instead of the direct use of formaldehyde during the synthesis. The use of HMT can effectively slow down the rate of reaction for the formation and self-polymerization of RUF, and thus provide the best possibility to guarantee the selective formation of RUF around the nucleation seeds (ZIF-8). Compared with the widely used approach in literature, which involves the dispersion of MOF precursors into the solution containing porous materials, our strategy, acting in an opposite way, presents a new option for design and synthesis of MOF-based core–shell composites.

In addition, through a simple carbonization of the obtained core–shell structured ZIF@RUF composite, an N-doped carbon material with microporous core and mesoporous shell structure, ZIFC@RUFC, can also be obtained. The sample (ZIFC@RUFC) exhibits significantly enhanced porosity including surface area, pore volume, and pore size, which improved CO_2_ capacity and diffusion rate than its individual counterparts of ZIFC and RUFC.

## Conflicts of interest

The authors declare no competing financial interest.

## Supplementary Material

RA-008-C8RA03349H-s001

## References

[cit1] Eddaoudi M., Kim J., Rosi N., Vodak D., Wachter J., O'Keefe M., Yaghi O. M. (2002). Systematic Design of Pore Size and Functionality in Isoreticular MOFs and Their Application in Methane Storage. Science.

[cit2] Kim H., Das S., Kim M. G., Dybtsev D. N., Kim Y., Kim K. (2011). Synthesis of Phase-Pure Interpenetrated MOF-5 and Its Gas Sorption Properties. Inorg. Chem..

[cit3] Zhao Z., Li Z., Lin Y. S. (2009). Adsorption and Diffusion of Carbon Dioxide on Metal–Organic Framework (MOF-5). Ind. Eng. Chem. Res..

[cit4] Dhakshinamoorthy A., Alvaro M., Corma A., Garcia H. (2011). Delineating Similarities and Dissimilarities in the Use of Metal Organic Frameworks and Zeolites as Heterogeneous Catalysts for Organic Reactions. Dalton Trans..

[cit5] Wang C., deKrafft K. E., Lin W. B. (2012). Pt Nanoparticles@Photoactive Metal–Organic Frameworks: Efficient Hydrogen Evolution *via* Synergistic Photoexcitation and Electron Injection. J. Am. Chem. Soc..

[cit6] Jayaramulu K., Kanoo P., George S. J., Maji T. K. (2010). Tunable Emission from a Porous Metal–Organic Framework by Employing an Excited-State Intramolecular Proton Transfer Responsive Ligand. Chem. Commun..

[cit7] Hendon C. H., Tiana D., Walsh A. (2012). Conductive Metal–Organic Frameworks and Networks: Fact or Fantasy?. Phys. Chem. Chem. Phys..

[cit8] Lee C. Y., Bae Y. S., Jeong N. C., Farha O. K., Sarjeant A. A., Stern C. L., Nickias P., Snurr R. Q., Hupp J. T., Nguyen S. T. (2011). Kinetic Separation of Propene and Propane in Metal–Organic Frameworks: Controlling Diffusion Rates in Plate-Shaped Crystals *via* Tuning of Pore Apertures and Crystallite Aspect Ratios. J. Am. Chem. Soc..

[cit9] Alaerts L., Kirschhock C., Maes M., Van der Veen M., Finsy V., Depla A., Martens J., Baron G., Jacobs P., Denayer J., De Vos D. (2007). Selective Adsorption and Separation of Xylene Isomers and Ethylbenzene with the Microporous Vanadium(iv) Terephthalate MIL-47. Angew. Chem., Int. Ed..

[cit10] James S. L. (2003). Metal–Organic Frameworks. Chem. Soc. Rev..

[cit11] Kaye S. S., Dailly A., Yaghi O. M., Long J. R. (2007). Impact of Preparation and Handling on the Hydrogen Storage Properties of Zn_4_O(1,4-benzenedicarboxylate)_3_ (MOF-5). J. Am. Chem. Soc..

[cit12] Low J. J., Benin A. I., Jakubczak P., Abrahamian J. F., Faheem S. A., Willis R. R. (2009). Virtual High Throughput Confirmed Experimentally: Porous Coordination Polymer Hydration. J. Am. Chem. Soc..

[cit13] Liu J., Wang Y., Benin A. I., Jakubczak P., Willis R. R., Levan M. D. (2010). CO_2_/H_2_O Adsorption Equilibrium and Rates on Metal–Organic Frameworks: HKUST-1 and Ni/DOBDC. Langmuir.

[cit14] Millward A. R., Yaghi O. M. (2005). Metal–Organic Frameworks with Exceptionally High Capacity for Storage of Carbon Dioxide at Room Temperature. J. Am. Chem. Soc..

[cit15] Llewellyn P. L., Bourrelly S., Serre C., Vimont A., Daturi M., Hamon L., Weireld G. D., Chang J. S., Hong D. Y., Hwang Y. K., Jhung S. H., Férey G. (2008). High Uptakes of CO_2_ and CH_4_ in Mesoporous Metal–Organic Frameworks MIL-100 and MIL-101. Langmuir.

[cit16] Furukawa H., Ko N., Go Y. B., Aratani N., Choi S. B., Choi E., Yazaydin A. O., Snurr R. Q., O'Keeffe M., Kim J., Yaghi O. M. (2010). Ultrahigh Porosity in Metal–Organic Frameworks. Science.

[cit17] Yazaydin A. O., Snurr R. Q., Park T. H., Koh K., Liu J., LeVan M. D., Benin A. I., Jakubczak P., Lanuza M., Galloway D. B., Low J. J., Willis R. R. (2009). Screening of Metal–Organic Frameworks for Carbon Dioxide Capture from Flue Gas Using a Combined Experimental and Modeling Approach. J. Am. Chem. Soc..

[cit18] Petit C., Burress J., Bandosz T. J. (2011). The Synthesis and Characterization of Copper-Based Metal–Organic Framework/Graphite Oxide Composites. Carbon.

[cit19] Prasanth K. P., Rallapalli P., Raj M. C., Bajaj H. C., Jasra R. V. (2011). Enhanced Hydrogen Sorption in Single Walled Carbon Nanotube Incorporated MIL-101 Composite Metal–Organic Framework. Int. J. Hydrogen Energy.

[cit20] Sudik A. C., Côté A. P., Wong-Foy A. G., O'Keeffe M., Yaghi O. M. (2006). A Metal–Organic Framework with a Hierarchical System of Pores and Tetrahedral Building Blocks. Angew. Chem., Int. Ed..

[cit21] Wu C. M., Rathi M., Ahrenkiel S. P., Koodali R. T., Wang Z. Q. (2013). Facile Synthesis of MOF-5 Confined in SBA-15 Hybrid Material with Enhanced Hydrostability. Chem. Commun..

[cit22] Chen X. C., Lukaszczuk P., Tripisciano C., Rümmeli M. H., Nazzal J. S., Pelech I., Kalenczuk R. J., Palen E. B. (2010). Enhancement of the Structure Stability of MOF-5 Confined to Multiwalled Carbon Nanotubes. Phys. Status Solidi B.

[cit23] Yang S. J., Choi J. Y., Chae H. K., Cho J. H., Nahm K. S., Park C. R. (2009). Preparation and Enhanced Hydrostability and Hydrogen Storage Capacity of CNT@MOF-5 Hybrid Composite. Chem. Mater..

[cit24] Anbia M., Hoseini V. (2012). Development of MWCNT@MIL-101 Hybrid Composite with Enhanced Adsorption Capacity for Carbon Dioxide. Chem. Eng. J..

[cit25] Pachfule P., Balan B. K., Kurungot S., Banerjee R. (2012). One-Dimensional Confinement of a Nanosized Metal Organic Framework in Carbon Nanofibers for Improved Gas Adsorption. Chem. Commun..

[cit26] Liu D., Purewal J. J., Yang J., Sudik A., Maurer S., Mueller U., Ni J., Siegel D. J. (2012). MOF-5 Composites Exhibiting Improved Thermal Conductivity. Int. J. Hydrogen Energy.

[cit27] Zhang Z. Z., Wang H., Chen X. Q., Zhu C. M., Wei W., Sun Y. H. (2015). Chromium-Based Metal–Organic Framework/Mesoporous Carbon Composite: Synthesis, Characterization and CO_2_ Adsorption. Adsorption.

[cit28] Rallapalli P. B. S., Raj M. C., Patil D. V., Prasanth K. P., Somani R. S., Bajaj H. C. (2013). Activated Carbon@MIL-101 (Cr): a Potential Metal–Organic Framework Composite Material for Hydrogen Storage. Int. J. Energy Res..

[cit29] Yu J., Mu C., Yan B. Y., Qin X. Y., Shen C., Xue H. G., Pang H. (2017). Nanoparticle/MOF composites: preparations and applications. Mater. Horiz..

[cit30] Zhu Q. L., Xu Q. (2014). Metal–organic framework composites. Chem. Soc. Rev..

[cit31] Ahmed I., Jhung S. H. (2014). Composites of metal–organic frameworks: preparation and application in adsorption. Mater. Today.

[cit32] Küsgens P., Siegle S., Kaskel S. (2009). Crystal Growth of the Metal–Organic Framework Cu_3_(BTC)_2_ on the Surface of Pulp Fibers. Adv. Eng. Mater..

[cit33] Buso D., Nairn K. M., Gimona M., Hill A. J., Falcaro P. (2011). Fast Synthesis of MOF-5 Microcrystals Using Sol–Gel SiO_2_ Nanoparticles. Chem. Mater..

[cit34] Xiang Z. H., Hu Z., Cao D. P., Yang W. T., Lu J. M., Han B. Y., Wang W. C. (2011). Metal–Organic Frameworks with Incorporated Carbon Nanotubes: Improving Carbon Dioxide and Methane Storage by Lithium Doping. Angew. Chem., Int. Ed..

[cit35] Furtado A. M. B., Liu J., Wang Y., Levan M. D. (2011). Mesoporous Silica-Metal Organic Composite: Synthesis, Characterization, and Ammonia Adsorption. J. Mater. Chem..

[cit36] Zhu C. M., Zhang Z. Z., Wang B. D., Chen Y. Y., Wang H., Chen X. Q., Zhang H. J., Sun N. N., Wei W., Sun Y. H. (2016). Synthesis of HKUST-1#MCF Compositing Materials for CO_2_ Adsorption. Microporous Mesoporous Mater..

[cit37] Yu J., Guo M. Y., Muhammad F., Wang A., Zhang F., Li Q., Zhu G. S. (2014). One-Pot Synthesis of Highly Ordered Nitrogen-Containing Mesoporous Carbon with Resorcinol–Urea–Formaldehyde Resin for CO_2_ Capture. Carbon.

[cit38] Park K. S., Ni Z., Côté A. P., Choi J. Y., Huang R., Uribe-Romo F. J., Chae H. K., O'Keeffe M., Yaghi O. M. (2006). Exceptional Chemical and Thermal Stability of Zeolitic Imidazolate Frameworks. Proc. Natl. Acad. Sci..

[cit39] Datta K. K. R., Balasubramanian V. V., Ariga K., Mori T., Vinu A. (2011). Highly Crystalline and Conductive Nitrogen-Doped Mesoporous Carbon with Graphitic Walls and Its Electrochemical Performance. Chem.–Eur. J..

[cit40] Zhang Z. Z., Zhao H. Y., Zhang L. N., Sun N. N., Wei W., Sun Y. H. (2017). One-Pot Solvent-Free Strategy for the Facile and Fast Synthesis of Highly Enriched Nitrogen-Doped Carbons. J. Phys. Chem. C.

[cit41] Ding S. M., Dong Q. L., Hu J. W., Xiao W. M., Liu X. H., Liao L. Q., Zhang N. (2016). Enhanced Selective Adsorption of CO_2_ on Nitrogen-Doped Porous Carbon Monoliths Derived from IRMOF-3. Chem. Commun..

[cit42] Liu D., Lei J. H., Guo L. P., Qu D. Y., Li Y., Su B. L. (2012). One-Pot Aqueous Route to Synthesize Highly Ordered Cubic and Hexagonal Mesoporous Carbons from Resorcinol and Hexamine. Carbon.

[cit43] Liu L., Xu S. D., Wang F. Y., Song Y. J., Liu J., Gao Z. M., Yuan Z. Y. (2017). Nitrogen-Doped Carbon Materials with Cubic Ordered Mesostructure: Low-Temperature Autoclaving Synthesis for Electrochemical Supercapacitor and CO_2_ Capture. RSC Adv..

[cit44] Zhang L. J., Su Z. X., Jiang F. L., Yang L. L., Qian J. J., Zhou Y. F., Li W. M., Hong M. C. (2014). Highly Graphitized Nitrogen-Doped Porous Carbon Nanopolyhedra Derived from ZIF-8 Nanocrystals as Efficient Electrocatalysts for Oxygen Reduction Reactions. Nanoscale.

[cit45] To J. W. F., He J. J., Mei J. G., Haghpanah R., Chen Z., Kurosawa T., Chen S. C., Bae W. G., Pan L. J., Tok J. B. H., Wilcox J., Bao Z. N. (2016). Hierarchical N-doped carbon as CO_2_ adsorbent with high CO_2_ selectivity from rationally designed polypyrrole precursor. J. Am. Chem. Soc..

[cit46] Liu L., Deng Q. F., Ma T. Y., Lin X. Z., Hou X. X., Liu Y. P., Yuan Z. Y. (2011). Ordered mesoporous carbons: citric acid-catalyzed synthesis, nitrogen doping and CO_2_ capture. J. Mater. Chem..

[cit47] Zhang Z. Z., Zhu C. M., Sun N. N., Wang H., Tang Z. Y., Wei W., Sun Y. H. (2015). One-pot solvent-free synthesis of nitrogen and magnesium codoped mesoporous carbon composites for CO_2_ capture. J. Phys. Chem. C.

[cit48] Feng S. S., Li W., Shi Q., Li Y. H., Chen J. C., Ling Y., Asiri A. M., Zhao D. Y. (2014). Synthesis of nitrogen-doped hollow carbon nanospheres for CO_2_ capture. Chem. Commun..

[cit49] Wei J., Zhou D. D., Sun Z. K., Deng Y. H., Xia Y. Y., Zhao D. Y. (2013). A controllable synthesis of rich nitrogen-doped ordered mesoporous carbon for CO_2_ capture and supercapacitors. Adv. Funct. Mater..

[cit50] Zhang Z. J., Huang S. S., Xian S. K., Xi H. X., Li Z. (2011). Adsorption Equilibrium and Kinetics of CO_2_ on Chromium Terephthalate MIL-101. Energy Fuels.

[cit51] Zhang Z. Z., Wang H., Li J. Y., Wei W., Sun Y. H. (2013). Experimental Measurements of the Adsorption Equilibrium and Kinetics of CO_2_ in Chromium-Based Metal–Organic Framework MIL-101. Adsorpt. Sci. Technol..

