# Supplementary material for: Facilely controlled synthesis of a core-shell structured MOF composite and its derived N-doped hierarchical porous carbon for CO2 adsorption
Source: RSC Adv. 2018 Jun 12;8(38):21460–71. doi: 10.1039/c8ra03349h (PMC9080950; doi:10.1039/c8ra03349h)
Supplement: RA-008-C8RA03349H-s001 [file RA-008-C8RA03349H-s001.pdf]

Electronic Supplementary Material (ESI) for RSC Advances.
This journal is © The Royal Society of Chemistry 2018

Supporting Information

Facilely Controlled Synthesis of Core-Shell Structured MOF Composite and Its Derived N-doped Hierarchical Porous Carbon for CO_2 Adsorption

Zhongzheng Zhang[a] , Nannan Sun[a,*], Wei Wei[a,b,*] and Yuhan Sun[a,b]

[a] CAS Key Laboratory of Low-Carbon Conversion Science and Engineering, Shanghai Advanced Research Institute, Chinese Academy of Science, Shanghai 201203, China

[b] School of Physical Science and Technology, Shanghai Tech University, Shanghai 201210, China

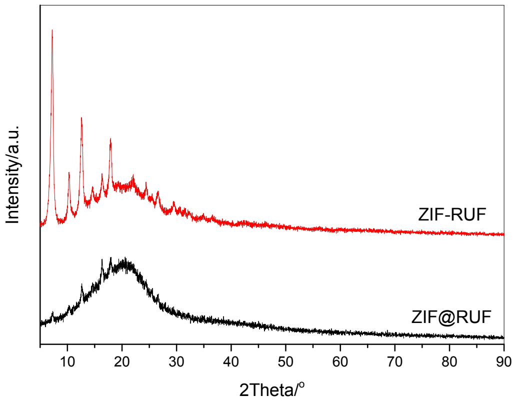

Fig.S1 XRD patterns of ZIF@RUF composite and ZIF-RUF physical mixture

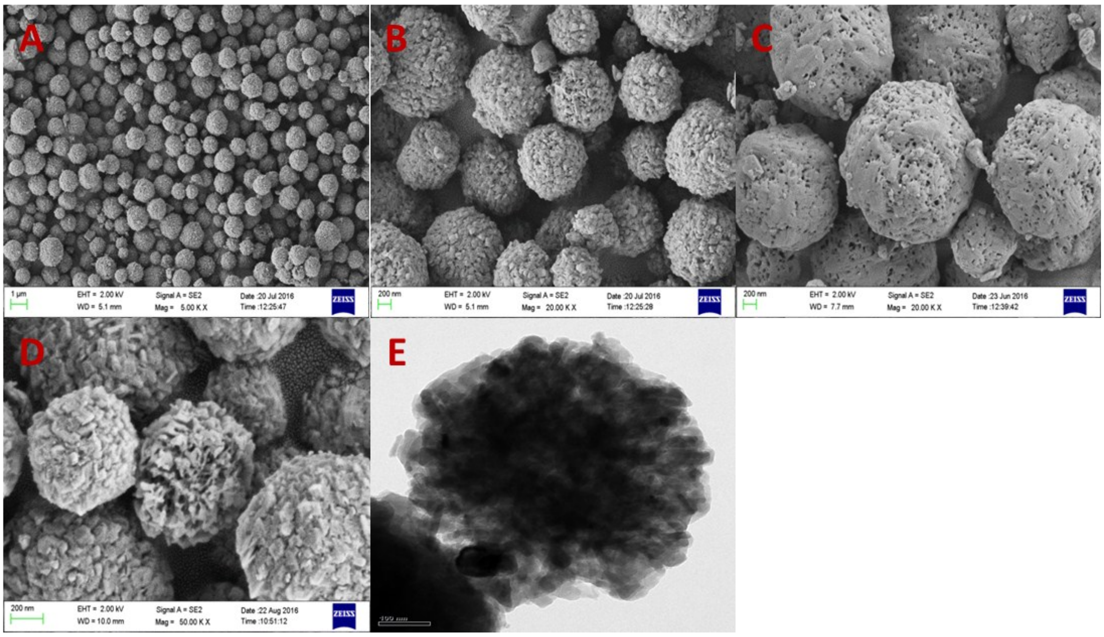

Fig.S2 SEM (A-D) and TEM (E) images of ZIF-8 particles

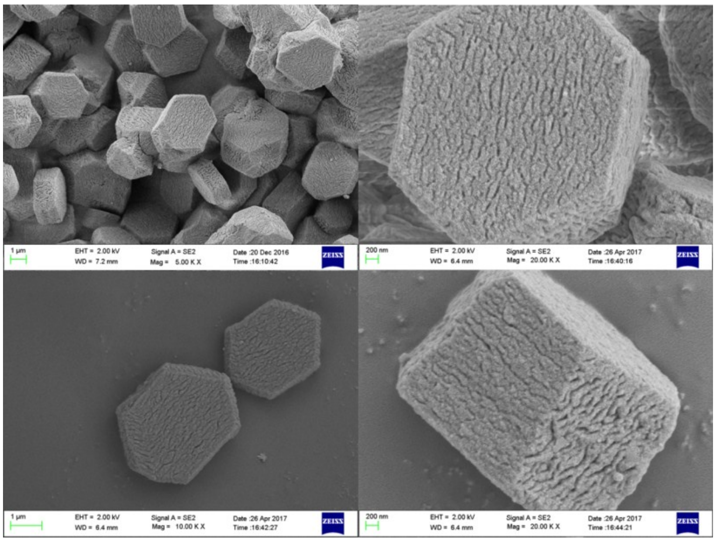

Fig.S3 SEM images of RUF sample

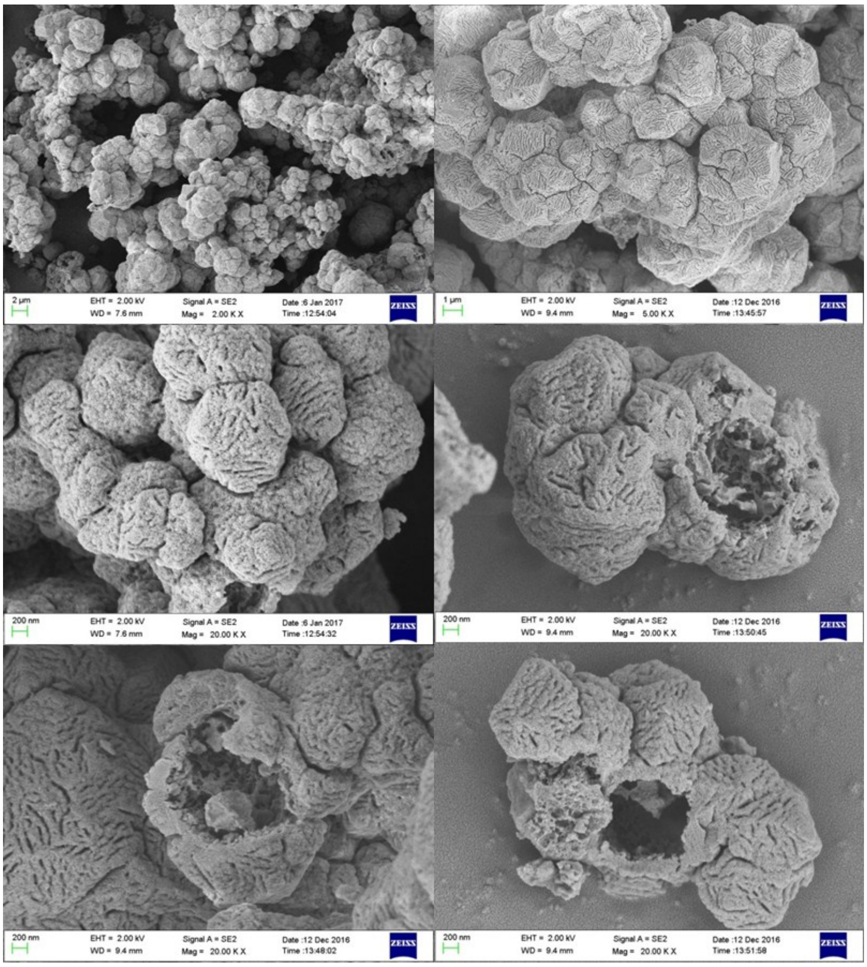

Fig.S4 SEM images of ZIF@RUF composite

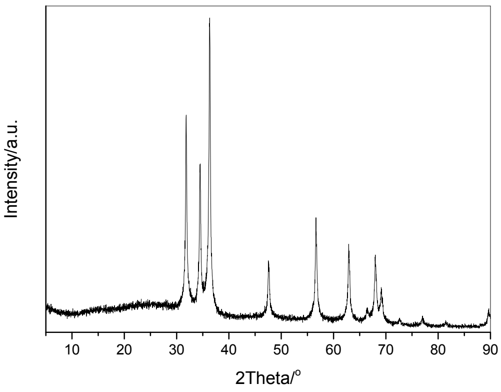

Fig.S5 XRD patterns of ZIFC-600 obtained by carbonization of ZIF-8 at 600 ^oC in N_2 atmosphere

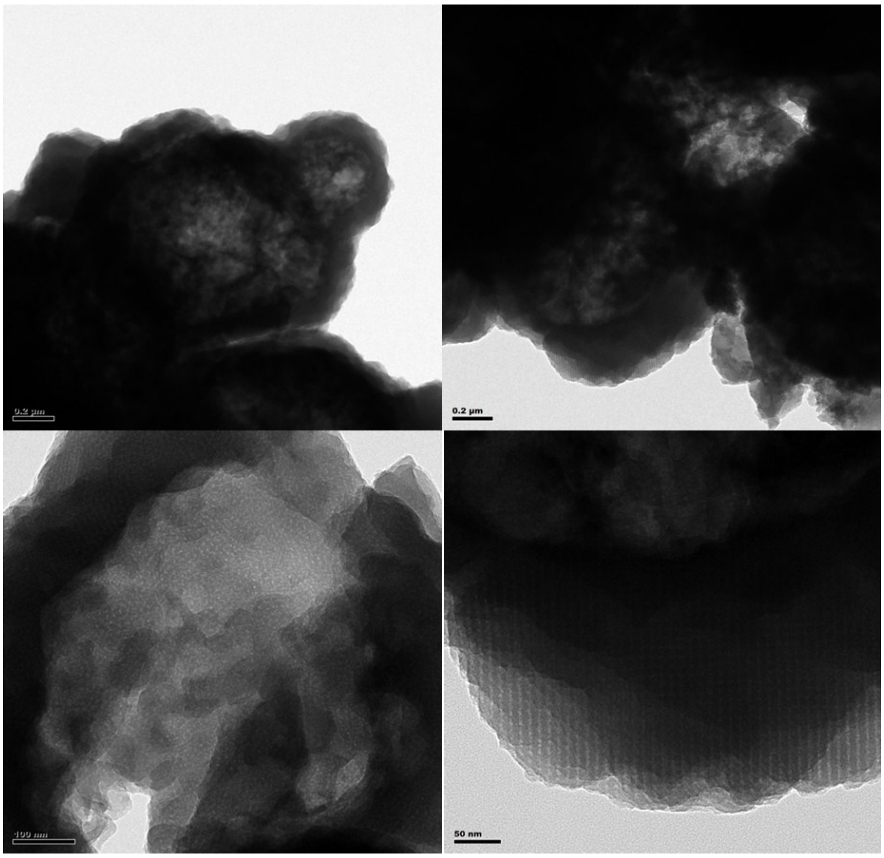

Fig.S6 TEM images of ZIFC@RUFC

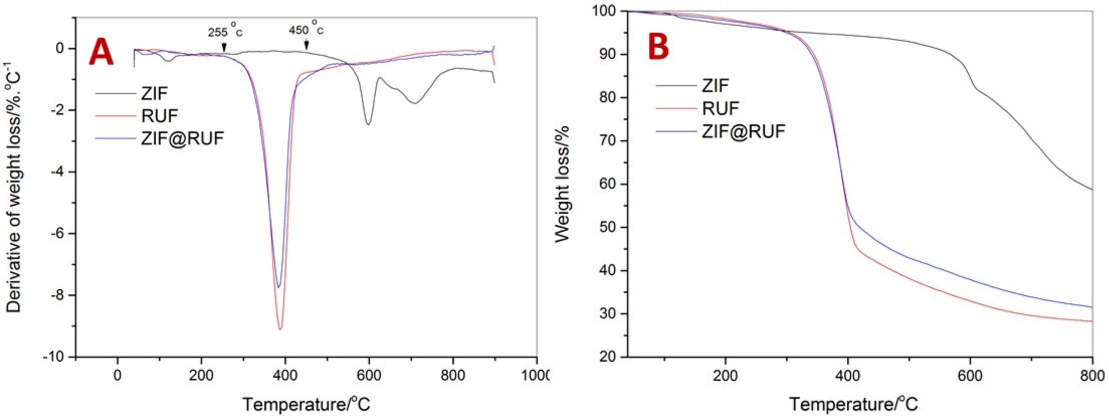

Fig.S7 Derivative thermogravimetric (A) and thermogravimetric analysis (B) of ZIF-8, RUF and ZIF@RUF under N_2 atmosphere

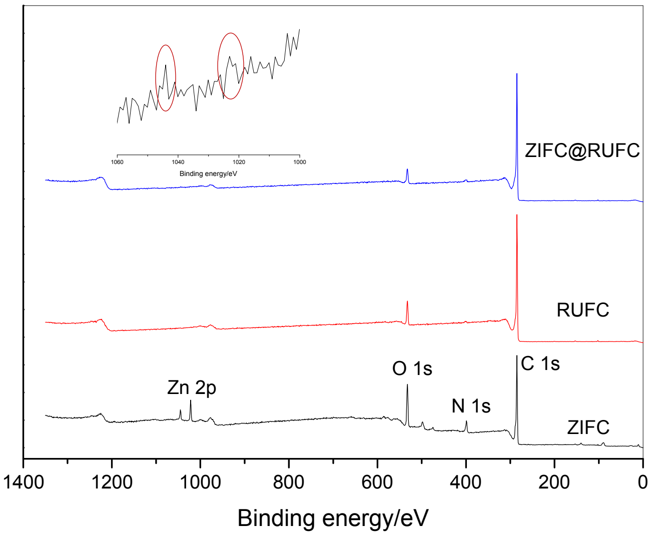

Fig.S8 XPS survey spectra of ZIFC, RUFC and ZIFC@RUFC. The inset is the magnified ZIFC@RUFC spectrum in the binding energy range of 1000-1060 eV.

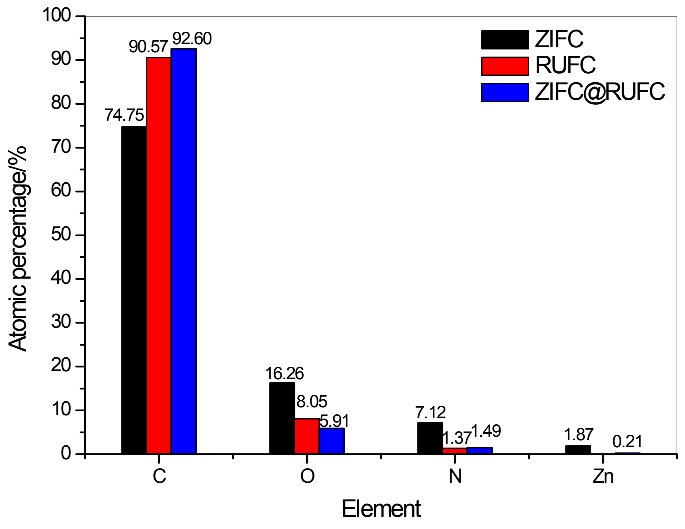

Fig.S9 C, N, O and Zn contents in ZIC, RUFC and ZIFC@RUFC samples from XPS

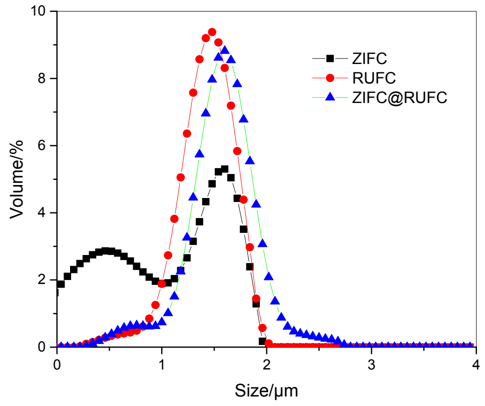

Fig.S10 Particle size distributions of ZIFC, RUFC, and ZIFC@RUFC sample
